# Case of Retroversion of the Uterus Terminating in Abortion and Death

**Published:** 1800

**Authors:** James Bell

**Affiliations:** one of the Physicians of the Kelso Dispensary; and formerly President of the Medical and Natural History Societies of Edinburgh.


IV. *Case of Retroversion of the Uterus terminating in Abortion and Death. Communicated in a letter to Dr.* Simmons, *by* James Bell, *M. D. one of the Physicians of the Kelso Dispensary; and formerly President of the Medical and Natural History Societies of Edinburgh.*

THE subject of this case was Jean Paton, a well shaped middle sized woman. She was in the 36th year of her age, and the mother of nine children. Having always been healthy during the period of gestation, her uneasiness on the present occasion was the more severely felt. She had suffered under her complaint for many weeks, before she applied for assistance from medical advice; and, as her circumstances were narrow, delicacy prevented her from incurring expences which she might be unable to discharge. Her sufferings however continuing unremitted, her husband at length resolved upon applying to me. He told me that as he lived at the distance of nine miles from my residence, he could could not requeſt me to viſit his wife; but that he hoped, by giving me a report of her ſituation, to enable me to order ſuch remedies as might tend to relieve her diſtreſs.

The report he made of the caſe was very incorrect and unſatisfactory. The chief circumſtances I could learn from him were, that his wife laboured under pain and difficulty in voiding urine, that ſhe believed herſelf to be with child, and that her general health was much impaired. The urinary ſymptoms had exiſted for ſeveral weeks, and had of late been greatly aggravated. Imperfect as this account was, a conſideration of the circumſtances which he ſtated, connected with the farther information that ſhe was yet in the early months of pregnancy, inclined me to be of opinion that her diſeaſe was a retroverſion of the uterus. But as the ſymptoms detailed did not furniſh grounds for a decided opinion, and as I was led to believe from the huſband's ſtatement, that there was no immediate urgency in the woman's ſituation, I contented myſelf with ordering the uſe of ſome laxative and diuretic remedies; ſtating to him, however, my apprehenſion, that his wife's complaints were of ſuch a nature as to demand every poffible care and attention. I at the fame time defired him to communicate to me, in a few days, the ftate in which the difeafe fhould then be, becaufe if the patient did not recover in that period, I fhould conceive it neceffary to vifit her. This injunction, however, had not the defired effect; and I heard nothing further of my patient until a week thereafter, when I received a meffage requefting me to fee her, as her fituation was now conceived to be very precarious. I accordingly faw her on Saturday, the 18th of Auguft, 1798, and unhappily found that my conjectures refpecting the nature of her complaint had been too well founded, and that there was much reafon to regret the latenefs of the period at which I had been called to adminifter relief.

She complained of a conftant and fevere pain in the region of the pelvis, which was greatly aggravated on paffing urine, and, in a lefs degree, by motion. The urine was voided in fmall quantity at a time, and with much difficulty, and there was no poffibility of voiding it except when fhe lay on her back; a pofture which fhe generally ufed from the greater eafe fhe experienced in it. When the urine was expelled ſhe always felt an alleviation of pain, and, as, on the contrary, the pain was greatly increaſed by any degree of diſtention of the bladder, ſhe was on this account under the neceſſity of paſſing it frequently.

She likewiſe complained of a conſiderable degree of pain over the abdomen, which was increaſed on preſſure, and was accompanied by much uneaſineſs and ſenſe of oppreſſion about the prœcordia, ſome degree of diſtention, more eſpecially about the epigaſtric region, frequent vomiting, and obſtinate conſtipation. She appeared languid and much debilitated, and her countenance indicated great diſtreſs. Her pulſe was ſmall, frequent, and feeble: her ſkin was moderately warm. She had no appetite, but her tongue was pretty clean, and ſhe did not complain of thirſt. She had not menſtruated for three months, and ſhe informed me that ſhe had every reaſon to believe ſhe had been pregnant for that period of time. The uterus however, had not riſen above the pubis, as appeared from there being no particular fulneſs in this region, much leſs the peculiar ſwelling of this organ when it has riſen above the brim of the pelvis.

 The

The urinary ſymptoms above deſcribed, had commenced in a few weeks after conception. They were for ſome time experienced but ſlightly, and appear to have conſiſted in a degree of ſtrangury, which never roſe to ſo great a height as to produce much uneaſineſs. This frequent attendant upon the early months of impregnation, put on a very different character about the middle of the ſecond month of her geſtation, immediately prior to which period ſhe had imprudently exerted herſelf in aſſiſting to remove her furniture from one houſe to another, and thus undergone a great deal of fatigue. At this period the pain and difficulty in voiding urine became much aggravated, and ſhe ſuffered a ſuppreſſion of urine for many hours. She now, likewiſe, firſt experienced the pain in the region of the pelvis, which was ſevere, conſtant, and much increaſed by the action of the bladder. This affection continued in a ſevere degree for ſome days, after which it gradually declined, though the urinary ſymptoms were much more diſtreſſing than they had been previous to this attack, and ſhe continued to feel in a greater or leſs degree the pain in the region of the pelvis.

During

During the ſeverity of her ſuffering, ſhe took ſome cream of tartar, and afterward a few laxative pills, which had the effect of keeping her eaſy in her bowels. Without the uſe of any other remedy, her complaints continued ſo moderate as to admit of her purſuing the leſs laborious parts of her houſehold occupation.

On the 8th of Auguſt, however, her ſtate of comparative eaſe was changed into intolerable agony, and, without any evident cauſe, her complaints were again aggravated in a very great degree. The pain in the region of the pelvis was much increaſed. The difficulty of paſſing urine was extreme. It was frequently ſuppreſſed for almoſt twenty-four hours, and, for ſome days after this ſecond attack, it was voided with ſuch intolerable pain, as to bedew her forehead with big drops of ſweat, producing great ſuffuſion of countenance, and ſometimes bringing on delirium. This extreme pain, and difficulty in voiding urine, gradually ſubſided; and for three or four days previous to that on which I ſaw her, it had been paſſed with more eaſe, and in greater quantity at a time.

The pain over the abdomen made its appearance in three days after the ſecond aggravation of her complaints, and had been increaſing in ſeverity. She had had no paſſage of her bowels for ſix days, and the vomiting had been preſent for nearly twenty-four hours.

The attendants had twice endeavoured to throw up a glyſter, but without ſucceſs.

Having acquired the above account of my patient, I had little doubt as to the nature of her ſituation. I therefore explained it to her, ſo far as was requiſite to induce her to ſubmit to the neceſſary meaſures for procuring relief, and proceeded to an examination per vaginam.

My finger ſoon reached a large, firm, round body, occupying the cavity of the pelvis, and I found, after a particular examination, that this body was the uterus in a ſtate of impregnation. The os uteri was turned upwards and forwards, and was nearly oppoſed to the ſuperior edge of the oſſa pubis. The fundus uteri was found reſting upon the ſacrum. The uterus was immoveable, and in a ſtate of complete retroverſion. One part of the examination deſerves notice. A large portion of the rectum was puſhed into the vagina, and protruded a conſiderable way beyond the external pudendum. The ſphincter ani was perfectly contracted, contracted, and the protruded gut might aptly be compared to a hernia deprived of its coverings. It formed, in fact, a true Elytrocele.

The examination having proved thus satisfactory in ascertaining the nature of the case, I proceeded to the execution of those measures of relief, which it appeared to me that the situation of my patient rendered necessary.

The urine, as I have already mentioned, had flowed more freely for some days, and there was no particular fulness in the hypogastric region. But although, from these circumstances, I had reason to believe there was no great quantity of urine in the bladder, yet, as the depletion of this viscus was indispensably necessary to the replacing of the uterus, I resolved on the use of the catheter to empty the bladder completely. I accordingly introduced the instrument with great ease, but without producing any considerable discharge of urine.

It was next endeavoured to throw up a glyster, but the uterus pressed the rectum so firmly against the posterior parietes of the pelvis, that the attempt was necessarily unsuccessful. I therefore proceeded to the operation for which thefe preliminary fteps had been employed, viz. the reduction of the uterus to its natural pofition.

Having introduced three fingers into the vagina, I placed them on different points of the uterus, and preffed this body upwards and forwards, fo as to raife it above the brim of the pelvis. The preffure I employed was very confiderable, but did not produce the fmalleft evident effect. Failing in this attempt, I refolved to place her on her knees with her fhoulders lowered, thus gaining the advantage of pofition. In this fituation it was again attempted to throw up a glyfter, but with no better fuccefs than before. I now renewed my attempts to reduce the uterus, and for this purpofe employed a degree of force, greater than that I had ufed in the former pofture. She complained but little, indeed greatly lefs than might have been expected. The preffure, however, excited ftrong efforts of bearing down, which were a confiderable bar to the fuccefs of the operation; and, at one time, when the uterus appeared to be giving way, and I had great hopes of fuccefs, the patient, by an involuntary forcible effort of this kind, deftroyed all the advantage I had gained. The motion and change of pofition having excited fevere and repeated attacks of vomiting, I thought it beft to abftain from farther efforts at this time, fatisfied that I had ufed every prudent degree of force in endeavouring to bring about the repofition of the uterus. But as I confidered the patient's fituation to be dangerous, and believed that upon the accomplifhment of this object her only chance of recovery depended, I did not defift without much regret, and refolved to renew my attempts next day with yet more freedom.

In the mean time fhe was ordered frequent fmall quantities of rich broths, and occafionally wine. I likewife directed for her a purgative bolus (compofed principally of Pulv. Jalap. gr. xv. and Hydr. Muriat. mit. gr. vi.) to be taken immediately, and to be repeated early next morning if it did not produce the defired effect. Her fituation being fuch as to give every reafon to believe that it would preclude the enjoyment of reft or fleep throughout the night, fhe was alfo ordered to have an opiate at bed-time, if her diftrefs continued urgent.

Such

Such was the fituation of my patient at the time of my vifit. She continued after I left her nearly in the fame ftate, until about eight o'clock in the evening, when fhe was feized with labour pains, and every fymptom of an approaching abortion. The labour pains were exceedingly moderate, and terminated, in a few minutes, in the expulfion of a healthy, well formed fœtus. The placenta immediately followed with little or no hemorrhage, and there was little additional diftrefs attendant upon the action of the uterus. There was, however, no alleviation of the pain, in the region of the pelvis, after the mifcarriage. On the contrary, fhe appears to have paffed a very reftlefs night.

Thefe circumftances were communicated to me early on the following morning by the huf-band, by whom I was alfo informed, that the change which had been produced in his wife's fituation by the abortion, had unfortunately in-duced the attendants to poftpone the ufe of the medicines I had prefcribed. I therefore enjoined him, immediately on his return home, to give directions that the purgative bolus fhould be adminiftered without delay, and that another attempt fhould be made to throw up an injection.

tion. The latter part of thefe directions alone was followed. An attendant endeavoured to adminifter the glyfter, but found an impoffibility of making it pafs on. The phyfic alfo was offered to her, as fhe had had no paffage of her bowels, but finding herfelf weak and exhaufted, fhe could not be prevailed on to take it.

She had little or no vomiting from the period of my leaving her. The ftate of debility, however, which was fo remarkable on that day, rapidly increafed, and about one o'clock in the afternoon of the 19th, her fufferings terminated in death.

The intereft produced by the circumftances of the cafe, induced me to ufe every exertion to obtain leave to infpect the body after death. I accordingly fucceeded in getting the better of the prejudices of the relatives, but not until the body had been deprived of life for nearly forty-eight hours. I was, however, happy to proceed to the diffection at any period, and was pleafed to find that the parts had undergone lefs change than there was reafon to have apprehended.

The abdomen was confiderably more diftended, and when a puncture was made through the the peritonæum into this cavity, a quantity of fœtid air ruſhed forth. When the opening in the peritonæum was further enlarged, the cavity of the abdomen was found likewiſe to contain about four pounds of an effuſed ſerous fluid, which was thick, turbid, and of a greeniſh colour. In this fluid there were many portions of coagulated gluten.

Having removed the effuſed ſerum, I proceeded to lay open the contents of the abdomen. I found a reſiſtance in turning back the divided coverings of the cavity, from a pretty firm and extenſive adheſion of the omentum to the peritonæum. Upon the deſtruction of this adheſion, ſtrong marks of inflammation were obſervable in different portions of the inteſtinal canal, more eſpecially in the great arch of the colon. The inferior orifice of the ſtomach was alſo much inflamed, and the inflammation extended for a conſiderable way upon the leſſer curve of this viſcus, and upon the duodenum. The inteſtines, particularly the larger, were conſiderably diſtended with flatus, and a great quantity of fœces was found in the colon. There was but little fœculent matter, however, below the ſigmoid flexure, and the principal congeſtion was about the caput coli, where where it was much hardened. The liver and kidneys did not partake of the inflammation.

Being ſatisfied with this part of the diſſection, I proceeded to the examination of the parts more immediately concerned in the diſeaſe.

On turning up the inteſtines, the bladder was diſcovered flaccid and much enlarged.— Here the inflammation had made greater progreſs. The peritonæum lining the bladder, and for a conſiderable diſtance around it, was covered with a ſtrong, thick, inflammatory cruſt. The coats of the bladder were every where much thickened, and upon the poſterior and ſuperior part of it, there was a portion of the ſize of half a crown in a ſtate of mortification. The diſſolution of the parts was ſo far advanced, that a very ſmall degree of violence produced an opening into the bladder. This opening was fully enlarged, but there was no farther appearance of ſphacelation, and no where any ſymptom of ſuppuration. There was very little urine in the cavity of the bladder. The uterus was found to have regained its natural poſition in the pelvis, and was almoſt reduced to its natural ſize. This organ appeared not to have ſuffered from inflammation, of which there were no marks, except in a very ſlight degree on the upper and back part of it. The protruded rectum, the ſtricture upon it having been removed, had alſo regained its natural ſituation.

Beſides thoſe above deſcribed, there were no appearances worthy of remark. The other cavities of the body were not opened.

*Obſervations.*—The caſe above related was for ſo ſhort a time under my particular notice, as to afford no room for multiplied obſervation. Nevertheleſs, the hiſtory of it is ſufficiently extended and circumſtantial, to render this inſtance of retroverſion of the uterus, not only intereſting but inſtructive. The whole circumſtances attending it forcibly point out the danger likely to ariſe from a neglect of the complaint, and ſo far as they are effects ariſing from a continued ſtate of retroverſion of the uterus, may be conſidered as applicable to every inſtance of the diſeaſe.

In attending to the hiſtory of this caſe, the firſt part of it which claims our particular notice, is the length of time during which the uterus was retroverted. It appears that the urinary ſymptoms put on no other character than that of a degree of dyſuria, until about ſix weeks previous to the death of the patient; when, when, after a ſuppreſſion of urine for ſome hours, the diſeaſe in queſtion was ſuperinduced. The retroverſion was productive of all the ſymptoms which commonly attend the diſeaſe. The fixed pain in the region of the pelvis and difficulty in voiding urine, were for many days diſtreſſing, and afterwards ſubſided into a ſtate of tolerable eaſe, notwithſtanding of the exiſtence of the retroverſion.

The diſeaſe having run this common courſe of exceſſive pain in the firſt inſtance, and conſequent alleviation, the unfortunate patient was lulled into a deluſive expectation of returning health, and unhappily truſted too implicitly to the exertions of unaſſiſted nature. Her diſeaſe, though quieſcent, continued to exiſt, and a renewed aggravation of her complaints was the obvious conſequence of her miſtaken conduct.

Upon this ſecond aggravation of ſymptoms, the ſtate of retroverſion moſt probably underwent a conſiderable change. It is reaſonable to ſuppoſe that during the five weeks in which it produced ſo little uneaſineſs, the retroverſion was not complete; but, on the contrary, that the degree of retroverſion which exiſted at the period of my examination, did not take place until until after the fuppreffion of urine, when the fundus of the uterus was depreffed from the fuperior part of the pelvis towards the inferior part of the facrum. I do not contend, however, that the aggravation of fymptoms might not have been induced by the increafe of the ovum, and confequent increafed preffure upon the bladder and parts concerned in the difeafe. But, of the two opinions, I confider the former as the moft probable, and that in the courfe of the complaint, the uterus fuffered different degrees of retroverfion.

Whatever was the caufe of the increafed difficulty in voiding urine and other attendant fymptoms, the change in the ftate of the complaint proved to be of the moft fatal nature. A violent inflammation immediately fucceeded to the fuppreffion of urine, and at the period when I vifited her, it had made the moft dreadful progrefs, having fpread rapidly over the abdominal vifcera. The appearances on diffection, fhow the irreparable ravages it had committed; and are conclufive, that in this inftance of retroverfion of the uterus, the primary difeafe was only the remote caufe of *inflammation*; and that it was this inflammation, together with the ftate of colic induced by the the mechanical preſſure of the uterus upon the rectum, which was the cauſe of death.

When the nature of the retroverſion of the uterus was firſt explained by the celebrated Doctor Hunter, it was conſidered to be a diſeaſe of great danger. It was believed, that in this ſituation of the uterus it muſt neceſſarily be liable to dangerous diſeaſe; and, that if it even eſcaped uninjured, it would be locked in the pelvis by the the gradual enlargement of the ovum, ſo that the repoſition of it would be altogether impracticable, and the death of the patient be a neceſſary event. The means of relief that were recommended, were of a nature commenſurate to the ſuppoſed magnitude of the danger.

A more extended experience, and a more particular obſervation of the appearances of the diſeaſe, have not confirmed the truth of theſe opinions; and medical practitioners have now ranked the complaint under the more innoxious claſs of female diſorders. To this opinion an eſteemed and judicious author has given his ſanction, and concludes his obſervations on this ſubject, by ſaying that "at the "preſent time, no practitioner of credit con- "ſiders it as a caſe of any difficulty, or feels

"any folicitude for the event, provided he be
"called to the relief of the patient before any
"mifchief is done."*

It has indeed been fhewn, that the uterus will fuftain no injury by its retroverfion, that there is no danger of its being locked in the pelvis, and that it will frequently be reftored by its own efforts to its natural pofition, even after the retroverfion has exifted for weeks. The modes of relief which are recommended, are therefore more lenient and lefs decifive: and we are advifed to content ourfelves with a careful attention to the urinary and alvine excretions, and whenever there is much refiftance to the repofition of the uterus by art, to truft for this defirable event to the exertions of nature.

As a general rule of practice, I confider this as entitled to the higheft confideration, and in the majority of cafes as adequate to the occafion. But there are fome inftances to which this rule is inadequate, and in which more decifive and active meafures only can refcue the patient from imminent danger. In

* Dr. Denman. Introduction to the Practice of Midwifery, vol. I. chap. iv.

this this clafs I conceive to be comprehended the cafe under confideration.

Since the attention of practitioners was fixed upon the retroverfion of the uterus, there have been many inftances of its fatal termination, and in thefe cafes "the death of the patient has been "difcovered to be owing to the injury done "the bladder only."* By which, I apprehend, we are to underftand, that inflammation of this vifcus had taken place, and that it had terminated partially in fuppuration or gangrene. This information is highly important, and its truth is corroborated by the appearances in the prefent inftance. The remarks which flow from it I fhall ftate as concifely as poffible.

The urinary bladder is fufceptible of various difeafes, and its organization and functions are deranged by no affection more than by inflammation. Amongft the various caufes which are capable of producing an inflammation in this part of the human frame, there is none of more certain operation than diftention. When this caufe is long continued therefore, and when it is conjoined with the mechanical fti-

* Introduction to the Practice of Midwifery. Loc. cit.

mulus of the preſſure of the uterus, the irritability of the blood veſſels of the bladder is exhauſted, and a ſtate of debility and conſequent inflammation are induced. The inflammation, whether it has begun in the proper coats of the bladder, or in the peritoneum, is ſoon communicated to this membrane, and is rapidly extended upon this general covering of the contents of the abdomen.

The conſequences of inflammation of the bladder muſt ever be a ſubject of much anxiety; and in retroverſion of the uterus, if we are to judge from the preſent inſtance, they will inſpire the moſt ſerious dread. Whether it may not ſometimes have been removed, I cannot pronounce; but this I may ſafely affirm, that there will be almoſt an impoſſibility of procuring a cure while the uterus is in a ſtate of retroverſion, or, perhaps, even when the reduction is readily effected, and every remedy is judiciouſly adminiſtered. It ſhould be our great object, therefore, to prevent the acceſſion of a ſympton, the removal of which is ſo very problematical, and which, if continued, will, ſo generally, terminate in death.

The judicious practitioner will carefully attend, then, to the ſtate of the bladder, and pay every attention to its frequent depletion. He will ſometimes ſucceed in preventing the diſtention, the cauſe of inflammation moſt particularly to be guarded againſt; but it is alſo true that he will often fail of ſucceſs, and that there have been caſes to which opportunity and ſkill contributed every aſſiſtance, where death followed the continuance of the retroverſion: and if there were a poſſibility of uſing the catheter regularly, when it is neceſſary to the depletion of the bladder, (a circumſtance which in the majority of caſes muſt be impoſſible) ſtill our apprehenſion muſt be alive while the preſſure of the uterus is continued, and while this preſſure is daily increaſed by the growth of the ovum. I apprehend, therefore, that if we are to be contented with mild remedies in thoſe caſes of retroverſion, where the ſymptoms are moderate, we are authorized in uſing the moſt active meaſures, whenever the diſtention of the bladder is conſiderable or long continued, and when the uterus, by its increaſe of ſize, occupies entirely the cavity of the pelvis, and produces great preſſure upon the bladder.

It will frequently happen that there will be great reſiſtance to the repoſition of the uterus, and ſo much force may be required as to endanger an abortion. In many ſituations which occur in our paſſage through life, we are neceſſitated to ſuffer a certain evil to procure a greater good. The accoucheur ſacrifices the life of the child to the ſafety of the mother; and here, likewiſe, morality will warrant us in hazarding the ſafety of the yet immature fœtus, when, by the acceſſion of ſo mortal a ſymptom as inflammation of the bladder, the life of an uſeful member of ſociety is endangered. The degree of riſk of a miſcarriage, ariſing from a free preſſure of the uterus, is not accurately aſcertained; but thus much is certain, that in ſeveral caſes, where erroneous views of danger produced perſevering and forcible efforts to procure the repoſition of the uterus, no abortion followed. In the preſent inſtance, the labour pains did not make their appearance till ſeveral hours after conſiderable preſſure had been employed, and had been perſevered in for a length of time.

In purſuing this line of conduct, therefore, it would appear, that at a very ſmall riſk of the ſafety of the fœtus, we can always have it in our power to relieve our patient from a painful and diſtreſſing complaint, and ſecure her her againſt the acceſſion of a mortal ſymptom: and we ſhall have this farther encouragement, that our attempts to reduce the retroverſion will not be productive of injury to the uterus itſelf; for of the many caſes which have been the ſubjects of manual ſkill, there is no inſtance that I know of, in which inflammation, or other bad conſequences, followed judicious force applied to this organ.

How far there is a poſſibility of the rectum being ſo firmly preſſed againſt the ſacrum by the retroverted uterus, as to produce a complete ſtricture of this gut, and the bladder eſcape uninjured, I do not pretend to ſay. If it ever does exiſt ſeparately, it will be another ſituation where the reduction of the retroverſion is abſolutely neceſſary. The ſtricture upon the rectum was ſo complete in the preſent inſtance, that even when it was endeavoured to remove it in ſome degree, by preſſing the uterus towards the pubis, there was no poſſibility of injecting the ſmalleſt quantity of liquid.

Such a ſituation left no room for doubt. I determined to relieve my patient of this certain cauſe of death, by replacing the uterus. That I did not ſucceed in the completion of my wiſhes, is to me no proof that the meaſures I adopted were erroneous, or that, in the majority of caſes, they would not be attended with the moſt beneficial effects.

